# Effect of Doping on Hydrogen Evolution Reaction of Vanadium Disulfide Monolayer

**DOI:** 10.1186/s11671-015-1182-y

**Published:** 2015-12-10

**Authors:** Yuanju Qu, Hui Pan, Chi Tat Kwok, Zisheng Wang

**Affiliations:** Institute of Applied Physics and Materials Engineering, Faculty of Science and Technology, University of Macau, Macao, SAR People’s Republic of China; Department of Electromechanical Engineering, Faculty of Science and Technology, University of Macau, Macao, SAR People’s Republic of China; College of Physics and Communication Electronics, Jiangxi Normal University, Nanchang, 330022 People’s Republic of China

**Keywords:** VS_2_ monolayers, Doping, Hydrogen production, Hydrogen evolution reduction, First-principles calculation

## Abstract

As cheap and abundant materials, transitional metal dichalcogenide monolayers have attracted increasing interests for their application as catalysts in hydrogen production. In this work, the hydrogen evolution reduction of doped vanadium disulfide monolayers is investigated based on first-principles calculations. We find that the doping elements and concentration affect strongly the catalytic ability of the monolayer. We show that Ti-doping can efficiently reduce the Gibbs free energy of hydrogen adsorption in a wide range of hydrogen coverage. The catalytic ability of the monolayer at high hydrogen coverage can be improved by low Ti-density doping, while that at low hydrogen coverage is enhanced by moderate Ti-density doping. We further show that it is much easier to substitute the Ti atom to the V atom in the vanadium disulfide (VS_2_) monolayer than other transitional metal atoms considered here due to its lowest and negative formation energy. It is expected that the Ti-doped VS_2_ monolayer may be applicable in water electrolysis with improved efficiency.

**Electronic supplementary material:**

The online version of this article (doi:10.1186/s11671-015-1182-y) contains supplementary material, which is available to authorized users.

## Background

Two-dimensional (2D) transitional metal dichalcogenide monolayers have received increasing attention because of their amazing physical, chemical, electronic, and magnetic properties [1–8]. The transition-metal dichalcogenides have the formula of MX_2_ (M is a transition metal element from groups IV to VI, and X is a chalcogen element), where one M-atom layer is sandwiched between two X-atom layers [1]. These 2D monolayers have been extensively investigated for possible applications in many areas of science and technology, from nanodevices, photoelectronics, catalysts, to the bioscience [9–17]. Their application as catalysts for hydrogen production in water electrolysis is particularly interesting because of their features, such as low cost, easy large-scale fabrication, and rich abundance on Earth [8, 11, 18–31]. Numerous studies have shown that the catalytic performance of 2D MX_2_ nanostructures is closely related to their conductivity and active sites at edges [18–33]. For example, the metallic edges of MoS_2_, such as the zigzag edge, are active for hydrogen evolution reaction (HER) in water electrolysis [28–32]. Metallic MX_2_ showed better catalytic activity than its semiconducting counterpart [21, 26]. To enhance the performance, the MoS_2_/graphene composite had been studied for HER because graphene may improve the conductivity and modify their morphologies [24, 27]. Recently, Pan reported that vanadium disulfide (VS_2_) monolayer shows the best HER performance in the considered systems and its catalytic activity depends on the hydrogen coverage during HER, which is reduced at high coverage due to the change of conductivity [19]. Kong et al. reported that doping is one of possible methods to improve their activity [28]. In this work, we investigate the effect of doping on the catalytic activity of the VS_2_ monolayer to improve the HER performance on the basis of first-principles calculation. A series of elements, including Ti, Nb, W, Ta, Mo, Pt, Fe, Co, and Ni, are systematically studied. We find that Ti is the best element to easily substitute V in the VS_2_ monolayer and improve the HER ability. We also show that the doping effect on HER strongly depends on the concentration of dopants.

## Methods

The design of catalysts for water electrolysis is based on the first-principles calculation. The hydrogen evolution reduction of the VS_2_ monolayer with the dopant is investigated to improve its catalytic ability. The Vienna Ab initio Simulation Package (VASP) [34] incorporated with the projector augmented wave (PAW) scheme [35, 36], which is based on the density functional theory (DFT) [37] and the Perdew-Burke-Ernzerhof generalized gradient approximation (PBE-GGA) [38], is used in our calculations. Supercells with lattices larger than 10 Å are used to investigate the doping effect and hydrogen-density-dependent HER ability. A 3 × 3 × 1 grid for k-point sampling, based on the Monkhorst and Pack scheme [39], for geometry optimization of supercells, and an energy cutoff of 450 eV are consistently used in our calculations. Densities of states (DOSs) are calculated based on a k-point sampling of 5 × 5 × 1. To avoid image-image interaction between two monolayers in neighboring supercells in the vertical direction, a vacuum region of at least 20 Å is used for separation. Good convergence is obtained with these parameters, and the total energy was converged to 2.0 × 10^−5^ eV/atom. Both of spin-unpolarized and spin-polarized calculations are carried out.

## Results and Discussion

**Fig. 1:**
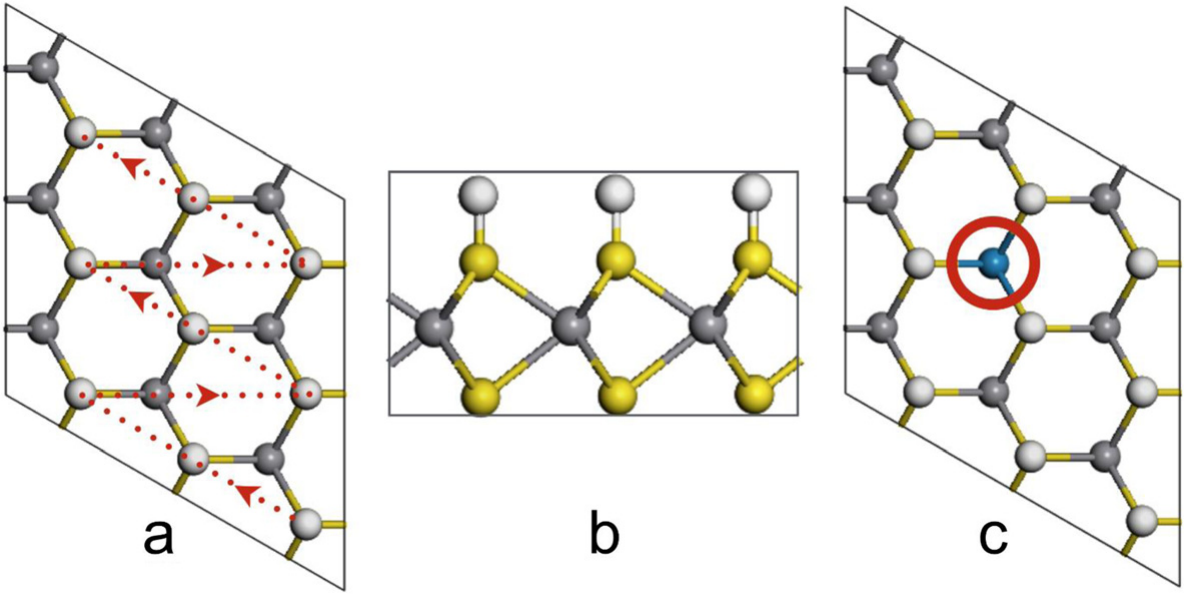
The representative structures of a fully H-covered VS_2_ 331 supercell: **a** top view, **b** side view, and **c** doped VS_2_ in the 331 supercell. The indication in (**a**) shows the way to take away the hydrogen atom one by one

In our calculations, a supercell with a hexagonal structure is set up on the basis of a unit cell of the VS_2_ monolayer with one surface fully covered by hydrogen atoms (*a* = 3.27 Å) [19] (Fig. 1 and Additional file 1: Figure S1), where the S–H bond length is about 1.37 Å. Two supercells with 3 × 3 × 1 and 4 × 4 × 1 unit cells (331 and 441 supercells, respectively) are used to investigate the effect of the doping concentration. Nine transition metal (TM) elements, including Ti, Nb, W, Ta, Mo, Pt, Fe, Co, and Ni, are considered as dopants in our calculations. To realize the doping, one or two V atoms in the supercells are substituted by TM atoms (Fig. 1c). The doped systems are fully relaxed to study the doping possibility and their HER performance.

Basically, the HER performance of the catalyst can be characterized by free energy of adsorption of reactive intermediates on its surface based on the Sabatier principle [40]. To qualify the catalytic ability, the reaction free energy of hydrogen adsorption (ΔG_H_) [19, 40–43] is calculated as the following equation:

1 $$ \Delta {\mathrm{G}}_{\mathrm{H}}=\Delta {\mathrm{E}}_{\mathrm{H}}+\Delta {\mathrm{E}}_{\mathrm{ZPE}}-\mathrm{T}\Delta {\mathrm{S}}_{\mathrm{H}} $$

where ΔE_H_ is the hydrogen chemisorption energy defined as:

2 $$ \Delta {\mathrm{E}}_{\mathrm{H}}=\mathrm{E}\left({\mathrm{VS}}_2+n\mathrm{H}\right)-\mathrm{E}\left({\mathrm{VS}}_2+\left(n-1\right)\mathrm{H}\right)-\frac{1}{2}\mathrm{E}\left({\mathrm{H}}_2\right) $$

where *n* is the number of H atoms adsorbed on a MX_2_ monolayer and changed from 1 to 9 (for full hydrogen coverage on the 331 supercell) (Fig. 1a, b) or 1 to 16 (for full hydrogen coverage on the 441 supercell) to investigate the effect of hydrogen coverage on catalytic activity. The hydrogen coverage refers to $\frac{n}{9}$ (in the 331 supercell) or $\frac{n}{16}$ (in the 441 supercell). Full coverage refers to each S atom on one side of the VS_2_ monolayer that is attached with one H atom. Therefore, ΔG_H_ as a function of the hydrogen coverage can be obtained. E(VS_2_ + *n*H), E(VS_2_), and E(H_2_) in Eq. (2) are the energies of the monolayer with hydrogen atoms (*n*), pure VS_2_ monolayer, and hydrogen molecule, respectively. ΔS_H_ is the difference in entropy. The entropy of adsorption of 1/2 H_2_ is $\Delta {\mathrm{S}}_{\mathrm{H}}\cong -1/2{\mathrm{S}}_{{\mathrm{H}}_2}^0$, where ${\mathrm{S}}_{{\mathrm{H}}_2}^0$ is the entropy of H_2_ in the gas phase at standard conditions. ΔE_ZPE_ is the difference in zero point energy between the adsorbed and the gas phase, related to the reaction 1/2H_2_(g) → H *, where H* denotes a hydrogen atom adsorbed on the surface. ΔE_ZPE_ − TΔS_H_ is about 0.24 eV [19, 40–43]. So, Eq. (1) is simplified to ΔG_H_ = ΔE_H_ + 0.24.

**Fig. 2:**
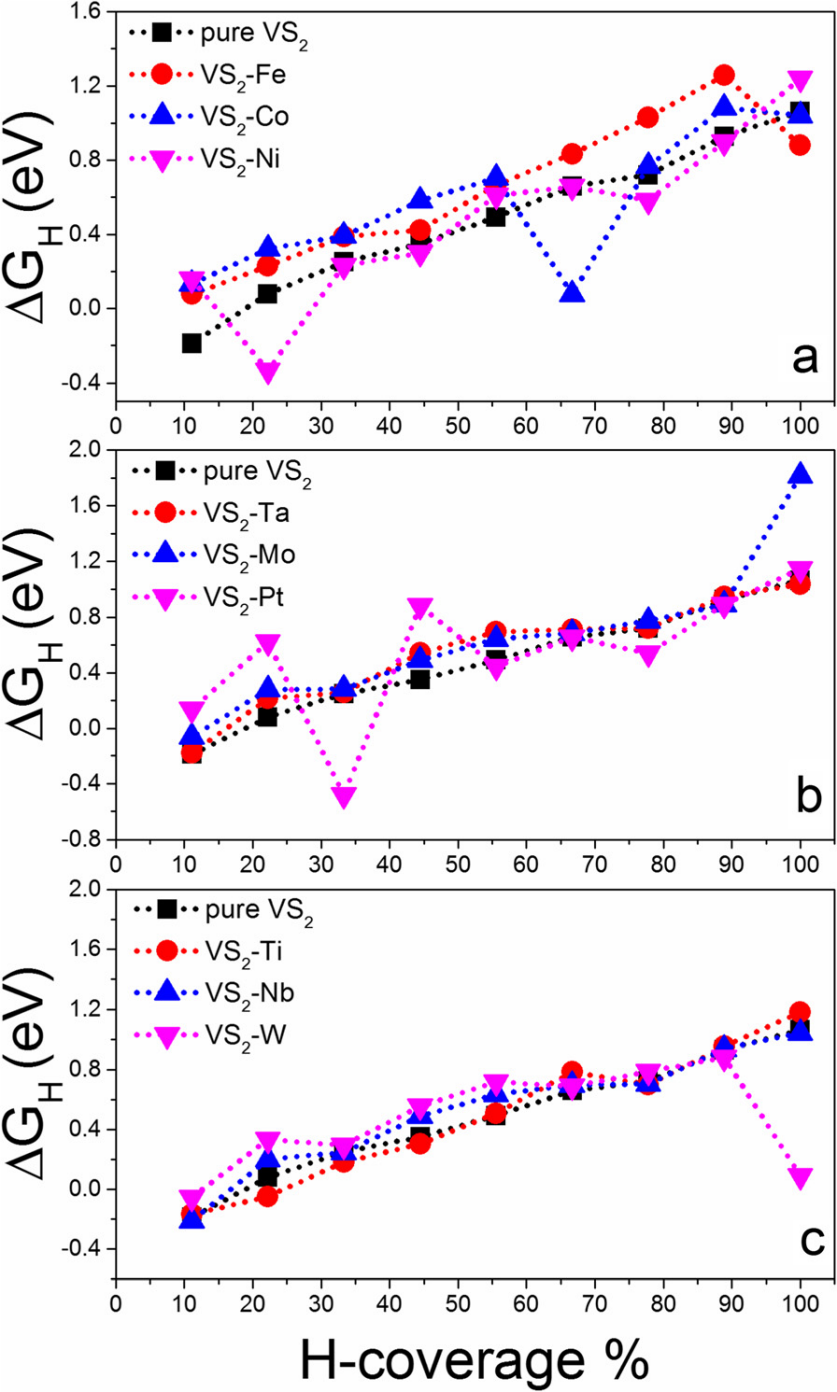
Calculated overpotentials as a function of H-coverage for TM-doped VS_2_ monolayers in 331 supercells with TM equal to: **a** Fe, Co, and Ni; **b** Ta, Mo, and Pt; and **c** Ti, Nb, and W. For comparison, the overpotentials of the pure VS_2_ 331 supercell are added

**Fig. 3:**
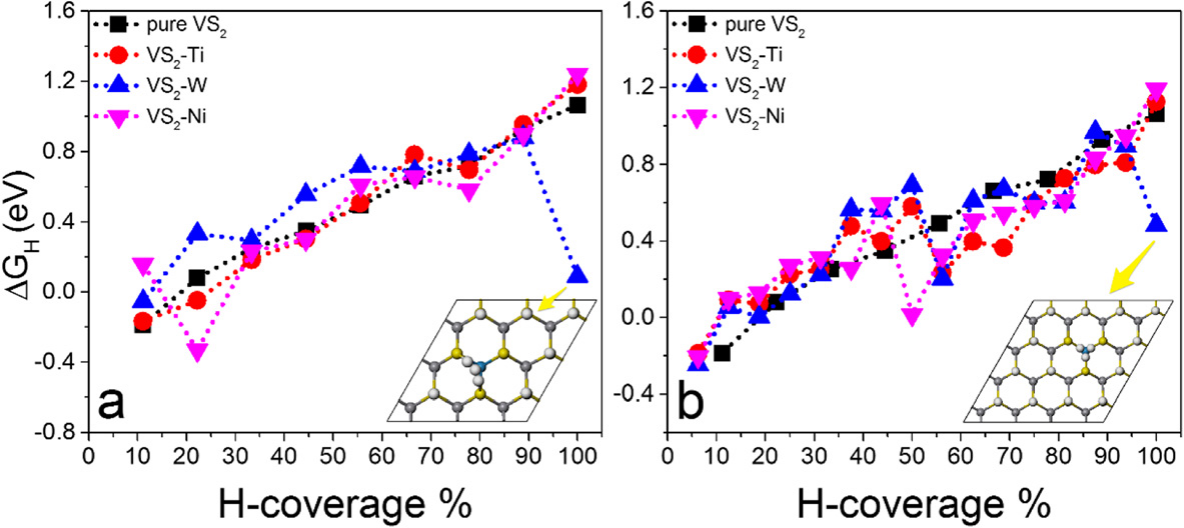
Calculated overpotentials as a function of H-coverage for the TM-doped VS_2_ (TM = Ti, W, Ni) monolayers in **a** 331 supercell and **b** 441 supercell

**Fig. 4:**
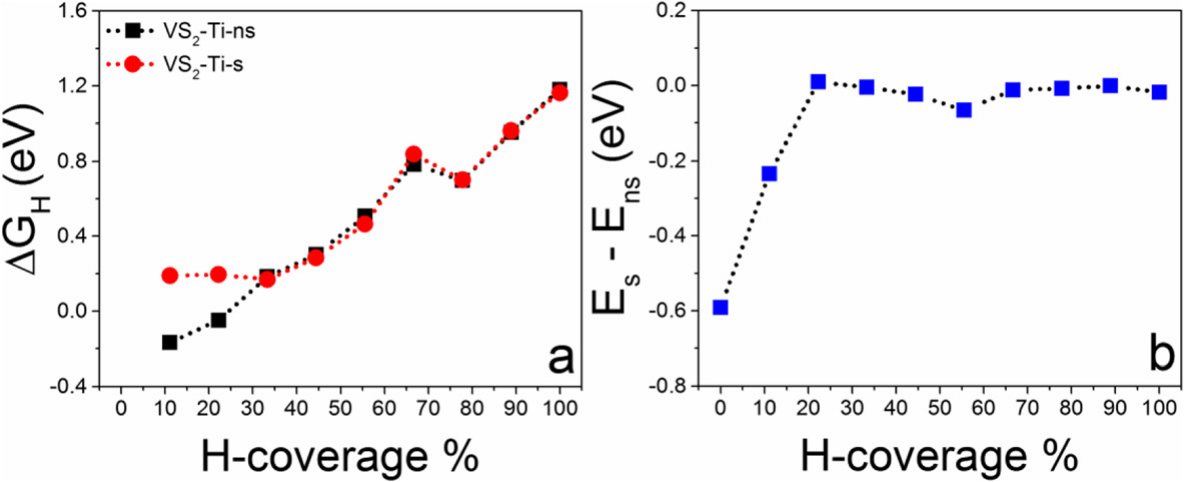
**a** Calculated overpotentials of Ti-doped VS_2_ in the 331 supercell with and without spin polarization. **b** Calculated energy difference between VS_2_-Ti with and without spin polarization, at various hydrogen coverages

To realize the partial hydrogen coverages, we start from the full hydrogen coverage on the supercell and take hydrogen atoms away one by one, as indicated in Fig. 1a. All of the systems with different hydrogen coverages are relaxed to calculate ΔG_H_. The relaxed structures show that their geometry are stable and hydrogen atoms keep on the tops of S atoms with the S–H bond of 1.37 Å (Additional file 1: Figure S2). We first study the 331 supercell with one V atom replaced by one TM atom, which is corresponding to a doping concentration of $\frac{1}{9}$. The calculated Gibbs free energies for hydrogen adsorption on the 331 supercell show that the catalytic activities of doped VS_2_ monolayers are still dependent on the hydrogen coverage, which decrease with the increment of hydrogen density (Fig. 2). Compared with a pure VS_2_ monolayer, we see that doping can partially improve its catalytic activity at a certain range of hydrogen density as indicated by the reduced ΔG_H_ (Fig. 2). For example, the Ni-doped VS_2_ monolayer shows better HER performance than a pure one in ranges of hydrogen density from $\frac{3}{9}\kern0.37em \mathrm{t}\mathrm{o}\;\frac{4}{9}$ and from $\frac{6}{9}\kern0.37em \mathrm{t}\mathrm{o}\;\frac{8}{9}$ (Fig. 2a). Pt-doping and Ti-doping improve the performance in hydrogen density ranging from $\frac{6}{9}\kern0.37em \mathrm{t}\mathrm{o}\;\frac{8}{9}$ (Fig. 2b) and from $\frac{1}{9}\kern0.37em \mathrm{t}\mathrm{o}\;\frac{5}{9}$ (Fig. 2c). Interestingly, we see that ΔG_H_ for W-doped VS_2_ at the full hydrogen coverage is almost close to zero (0.09 eV) (Fig. 2c). In all of the considered doping elements, we see that Ni- and Ti-doping can improve the HER performance of the VS_2_ monolayer in a wide hydrogen coverage and W-doping can dramatically enhance its catalytic activity at a high hydrogen coverage. For comparison, we put the calculated ΔG_H_ of Ni-, Ti-, and W-doped VS_2_ monolayers as a function of hydrogen density together (Fig. 3a). Clearly, Ti-doping is better than Ni-doping on the HER performance in a range of $\frac{1}{9}\kern0.37em \mathrm{t}\mathrm{o}\;\frac{5}{9}$, while Ni-doping is better than Ti-doping in a range of $\frac{6}{9}\kern0.37em \mathrm{t}\mathrm{o}\;\frac{8}{9}$ (Fig. 3a). W-doping is the best at the full hydrogen coverage. The relaxed structure of the W-doped VS_2_ monolayer with full hydrogen coverage shows that one of the H atoms moves away from the surface around the W-doping site and bonds to other H atoms nearby (inset in Fig. 3a), where the S–H and H–H bond lengths are 1.662 and 0.983 Å, respectively. We also investigate the effect of spin-polarization on the HER performance of the Ti-doped system. We find that spin-polarization may affect slightly the calculated Gibbs free energy at a lower hydrogen coverage but is negligible as the hydrogen coverage increases (Fig. 4). Therefore, spin-polarization is not considered below.

To investigate the effect of the doping concentration on the HER performance, the increment and reduction of the doping density are achieved by replacing more V atoms in the same supercell and enlarging the size of the supercell, respectively. For example, we replace two V atoms using two TM (Ti, Ni, or W) atoms in the 331 supercell, which is equivalent to a doping density of 22.22 % $\left(\frac{2}{9}\right)$. The calculated Gibbs free energies show that increasing the doping density makes the HER performance worse, indicating that a high doping concentration is not good in application (Additional file 1: Figure S3). Then, we use a 441 VS_2_ supercell with one V substituted by one TM atom (TM = Ti, Ni, and W), which equals to a doping density of about 6.25 % $\left(\frac{1}{16}\right)$. Clearly, the TM-doping can improve the HER performance of the VS_2_ monolayer under high-H coverage in a range of $\frac{8}{16}\;\mathrm{t}\mathrm{o}\;\frac{16}{16}$ (Fig. 3b). The catalytic abilities of Ti- and Ni-doped systems are enhanced by 50 % in the hydrogen coverage from $\frac{8}{16}\;\mathrm{t}\mathrm{o}\;\frac{11}{16}$ (Fig. 3b). However, Ni-doping reduces its performance in hydrogen coverages less than $\frac{8}{16}$. The catalytic ability of the Ti-doped VS_2_ monolayer at low hydrogen coverages is comparable with or slightly worse than that of the pure VS_2_ monolayer. Similarly, W-doping in the 441 supercell can only improve the HER performance at certain hydrogen densities, such as $\frac{9}{16}\;\mathrm{and}\;\frac{16}{16}$ (Fig. 3b). The relaxed structure of W-doped VS_2_ with the full hydrogen coverage shows that hydrogen atoms around the doping site move together to form a triangle with a H–H bond length of 1.00 Å and an extended S–H length of 1.90 Å (inset in Fig. 3b). From the calculated Gibbs free energies, we see that Ti is the best candidate as a dopant to improve the HER performance of the VS_2_ monolayer. Comparing with other MX_2_ monolayers, we see that the basal plane of the Ti-doped VS_2_ monolayer has better catalytic performance under the same condition (ΔG_H_ = −0.19 eV at 6.25 % coverage) than that of pure 1T-WS_2_ (ΔG_H_ = 0.28 eV) [21]. It is also worth noting that after doping Ti, Ni, and W atoms individually into the VS_2_ monolayer, the catalytic ability of the basal plane on the doped VS_2_ monolayer has improved dramatically at certain hydrogen coverages, which are equivalent to or even better than that of the active edges sites of MoS_2_, MoSe_2_, WS_2_, and WSe_2_ under the same hydrogen coverages [20]. For example, the catalytic activity of the Ti-doped VS_2_ monolayer (its ΔG_H_ equals −0.05 eV under a hydrogen coverage of $\frac{2}{9}$ (22.2 %)) is comparable to or better than that at the edges of MoS_2_, MoSe_2_, WS_2_, and WSe_2_ (the optimal ΔG_H_ is from −0.06 to 0.06 eV under a hydrogen coverage of 25 %).

**Fig. 5:**
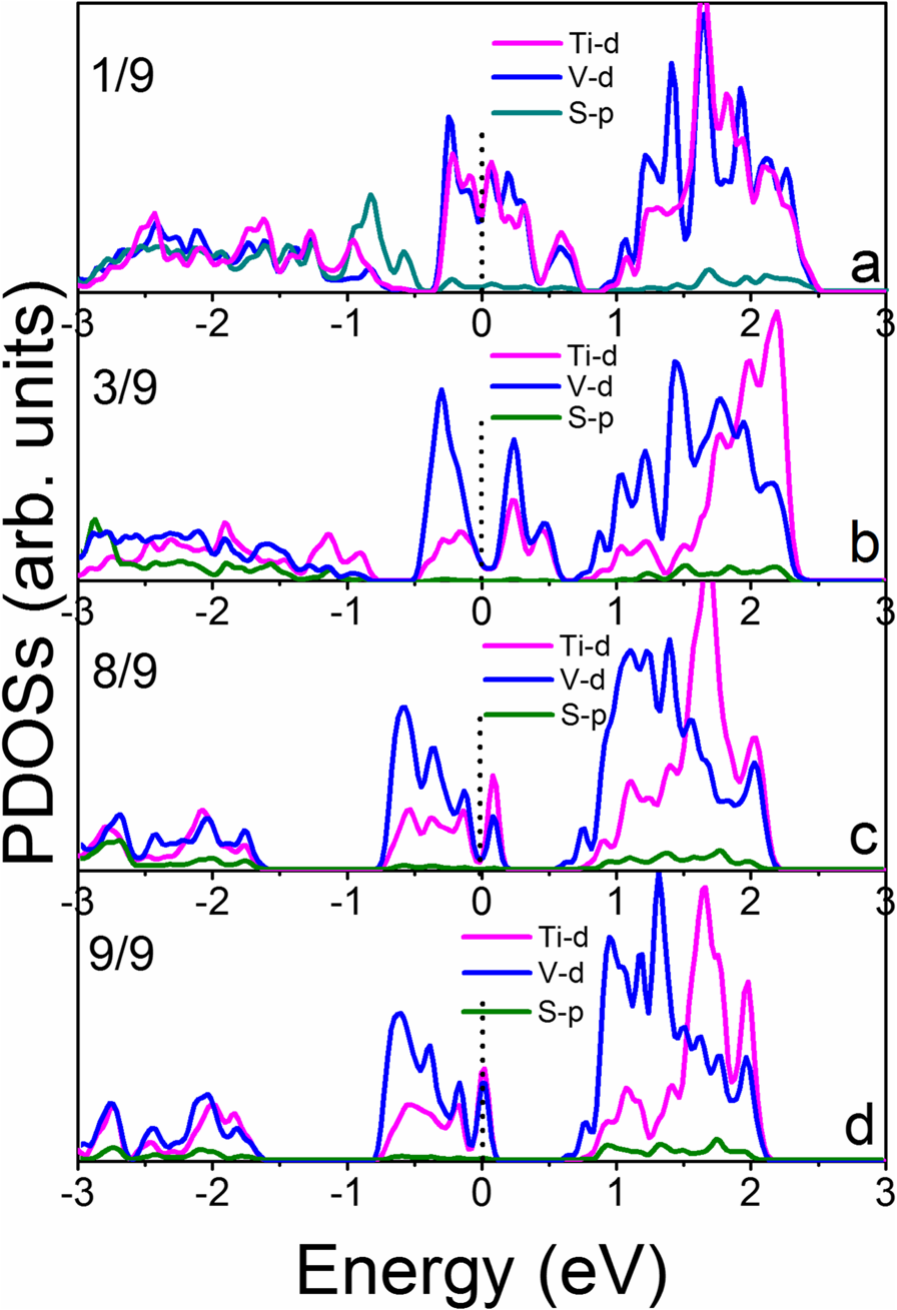
Calculated partial density of states of the Ti-doped VS_2_ monolayer in the 331 supercell with a hydrogen coverage at: **a** 1/9, **b** 3/9, **c** 8/9, and **d** 9/9

**Fig. 6:**
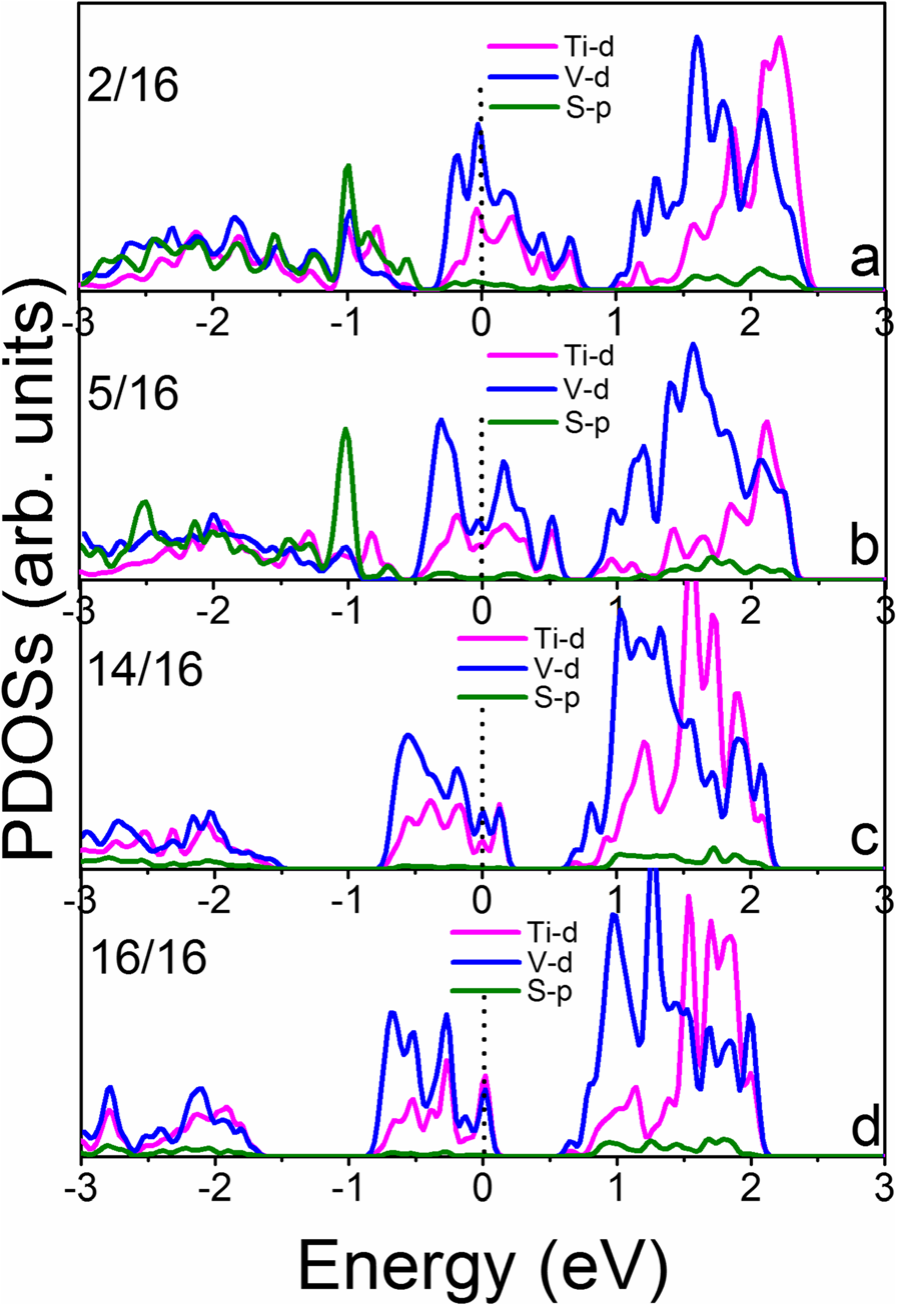
Calculated partial density of states of the Ti-doped VS_2_ monolayer in 441 supercell with a hydrogen coverage at: **a** 2/16, **b** 5/16, **c** 14/16, and **d** 16/16

To reveal the mechanism of the doping effect on HER performance, the partial density of states is calculated and shows the shift of the Fermi level as the hydrogen coverage increases (Figs. 5 and 6; Additional file 1: Figures S4–S7). We see that the densities of states of the Ti-doped VS_2_ monolayer under various hydrogen coverages near Fermi levels are mainly contributed to Ti-d and V-d electrons (Figs. 5 and 6). In the 331 supercell, localized defect states near the Fermi level are formed at high hydrogen coverage (Fig. 5c, d), which may lead to reduced carrier mobility and HER performance (Fig. 3a). However, the doping states near the Fermi level in the 441 supercell are connected to the valence band (Fig. 6c, d), resulting in better carrier mobility and improved catalytic activity at high hydrogen coverages (Fig. 3b).

**Fig. 7:**
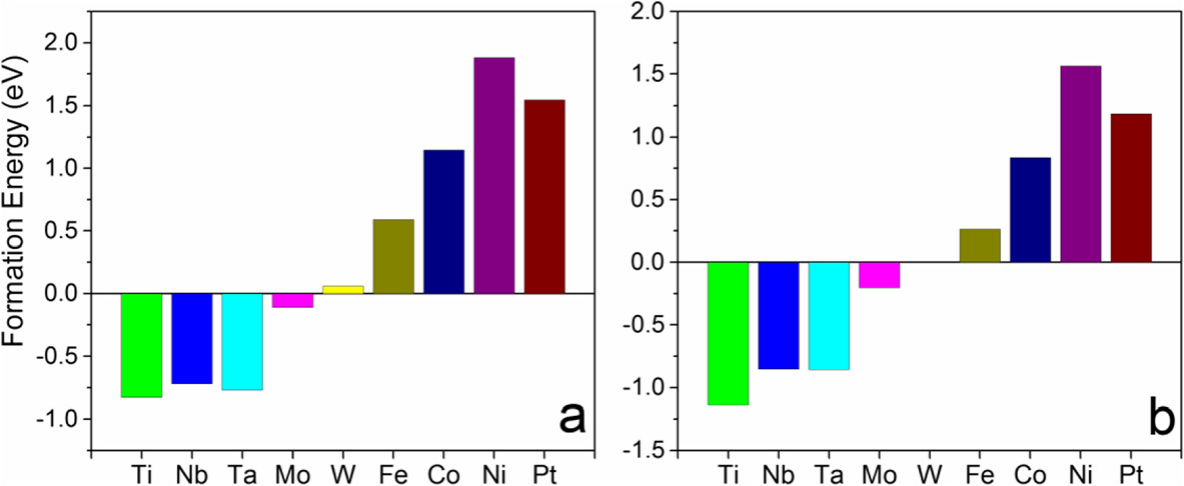
Calculated formation energies of the TM-doped VS_2_ monolayers (TM = Ti, Nb, Ta, Mo, W, Fe, Co, Ni, Pt) in **a** 331 supercell and **b** 441 supercell

Although the calculated Gibbs free energies show that Ti-doping may improve the HER performance of the VS_2_ monolayer, another important issue, which is the doping ability, needs to be stated. The possibility for the dopant to substitute the V atom in the host can be investigated by calculating the formation energy as below:

3 $$ {\mathrm{E}}_f=\left(\mathrm{E}\left({\mathrm{V}\mathrm{S}}_2+n\mathrm{T}\mathrm{M}\right)-\mathrm{E}\left({\mathrm{V}\mathrm{S}}_2\right)-n{\mu}_{\mathrm{TM}}+n{\mu}_{\mathrm{V}}\right)/n $$

where E(VS_2_ + *n*TM) and E(VS_2_) are the total energies of VS_2_ monolayer supercells with and without dopants. *μ*_TM_ and *μ*_V_ are the energies of TM and V atoms, respectively. *n* is the number of dopants in each supercell (*n* = 1). Our calculations show that the formation energies for Ti-, Mo-, Nb-, and Ta-doping are negative, indicating the reactions are exothermic, while the doping of W, Fe, Co, Ni, and Pt are endothermic because of their positive formation energies (Fig. 7). Particularly, it is easy to substitute the Ti atom to the V atom in the 331 supercell of the VS_2_ monolayer because of its lowest formation energy (−0.83 eV) (Fig. 7a). W-doping may be achieved under suitable conditions because its endothermic energy is as low as 0.06 eV. Compared with other elements, however, Ni-doping should be difficult because large energy is required (E_*f*_ = 1.88 eV). We further see that their formation energies are reduced if the doping density decreases (Fig. 7b). In this case, the energies of Ti- and W-substitutions are reduced to −1.14 and 0.005 eV, respectively, at a doping density of 6.25 % $\left(\frac{1}{16}\right)$ (Fig. 7b), indicating that doping at a low concentration is easier than that at a high concentration.

## Conclusions

We present a first-principles study on the effect of doping on the hydrogen evolution reaction of the VS_2_ monolayer. We find that the catalytic activity of the doped VS_2_ monolayer depends strongly on the choice of dopant and the doping concentration. The catalytic ability of the VS_2_ monolayer under high hydrogen coverages can be dramatically enhanced by TM-doping at a low concentration, while that under low hydrogen coverages can be improved by the doping at a moderate density. High-density doping results in reduced HER activity. We further show that Ti-doping should be the best to improve the HER ability of VS_2_ monolayers in our considered doping elements because of the reduced Gibbs free energy at a wide range of hydrogen coverages. By investigating the formation energy of TM substitution of the V atom, we find that the reaction of Ti-substitution of V in the VS_2_ monolayer is exothermic and easier than other TM elements due to its lowest formation energy. It is predicted that the Ti-doped VS_2_ monolayer may show better HER performance and find applications as catalysts in water electrolysis.

## Additional file

Additional file 1: Figure S1. Relaxed pure VS_2_ monolayer unit cell: (a) top view, (b) side view, relaxed VS_2_ monolayer unit cell with one side fully hydrogenated: (c) top view, (d) side view. **Figure S2.** Relaxed 331 supercells of VS_2_-Ti-doped from pure to full hydrogen coverages: (a) 0/9 or pure, (b) 1/9, (c) 2/9, (d) 3/9, (e) 4/9, (f) 5/9, (g) 6/9, (h) 7/9, (i) 8/9, (j) 9/9 or full hydrogen coverage. **Figure S3.** Calculated overpotentials as a function of H-coverage of VS_2_ with 3 dopant (Ti, W, Ni) atoms in 331 supercells. **Figure S4.** Calculated partial density of states of various H-covered VS_2_-W monolayer in 331 supercell with a hydrogen coverage at: (a) 1/9, (b) 3/9, (c) 8/9, and (d) 9/9. **Figure S5.** Calculated partial density of states of various H-covered VS_2_-W monolayer in 441 supercell with a hydrogen coverage at: (a) 2/16, (b) 5/16, (c) 14/16, and (d) 16/16. **Figure S6.** Calculated partial density of states of various H-covered VS_2_-Ni monolayer in 331 supercell with a hydrogen coverage at: (a) 1/9, (b) 3/9, (c) 8/9, and (d) 9/9. **Figure S7.** Calculated partial density of states of various H-covered VS_2_-Ni monolayer in 441 supercell with a hydrogen coverage at: (a) 2/16, (b) 5/16, (c) 14/16, and (d) 16/16. (DOC 1241 kb)
