# Supplementary material for: Effect of Doping on Hydrogen Evolution Reaction of Vanadium Disulfide Monolayer
Source: Nanoscale Res Lett. 2015 Dec 10;10:480. doi: 10.1186/s11671-015-1182-y (PMC4675759; doi:10.1186/s11671-015-1182-y)
Supplement: Additional file 1: Figure S1. — Relaxed pure VS2 monolayer unit cell: (a) top view, (b) side view, relaxed VS2 monolayer unit cell with one side fully hydrogenated: (c) top view, (d) side view. Figure S2. Relaxed 331 supercells of VS2-Ti-doped from pure to full hydrogen coverages: (a) 0/9 or pure, (b) 1/9, (c) 2/9, (d) 3/9, (e) 4/9, (f) 5/9, (g) 6/9, (h) 7/9, (i) 8/9, (j) 9/9 or full hydrogen coverage. Figure S3. Calculated overpotentials as a function of H-coverage of VS2 with 3 dopant (Ti, W, Ni) atoms in 331 supercells. Figure S4. Calculated partial density of states of various H-covered VS2-W monolayer in 331 supercell with a hydrogen coverage at: (a) 1/9, (b) 3/9, (c) 8/9, and (d) 9/9. Figure S5. Calculated partial density of states of various H-covered VS2-W monolayer in 441 supercell with a hydrogen coverage at: (a) 2/16, (b) 5/16, (c) 14/16, and (d) 16/16. Figure S6. Calculated partial density of states of various H-covered VS2-Ni monolayer in 331 supercell with a hydrogen coverage at: (a) 1/9, (b) 3/9, (c) 8/9, and (d) 9/9. Figure S7. Calculated partial density of states of various H-covered VS2-Ni monolayer in 441 supercell with a hydrogen coverage at: (a) 2/16, (b) 5/16, (c) 14/16, and (d) 16/16. (DOC 1241 kb) [file 11671_2015_1182_MOESM1_ESM.doc]

Effect of Doping on Hydrogen Evolution Reaction of Vanadium Disulfide Monolayer

Supporting Data

Yuanju Qu^1,2,3^, Hui Pan^1^*, Chi Tat Kwok^2^, and Zisheng Wang^3,1^

^1^Institute of Applied Physics and Materials Engineering, Faculty of Science and Technology, University of Macau, Macao SAR, P. R. China

^2^ Department of Electromechanical Engineering, Faculty of Science and Technology, University of Macau, Macao SAR, P. R. China

^3^ College of Physics and Communication Electronics, Jiangxi Normal University, Nanchang 330022, P. R. China

Results and Discusion

In one unit cell of VS_2_ monolayer that only contains one vanadium atom and two sulfur atoms, the lattice parameters are a=b=3.17 Å, after fully hydrogen adsorption on one surface of VS_2_ monolayer, the lattice parameters are a=b=3.27 Å, from which the fully hydrogen covered VS_2_ 331 supercells are constructed (S1). The relaxed topological structures (331 supercells) of VS_2_-Ti-doped from pure to fully covered by hydrogen atoms are shown in S2. No obvious distortions have been reserved, the bond length of H-S varies from 1.365 Å to 1.371 Å, which is less than 0.25% variation from its average bond length (1.368 Å). This suggests good stabilities of hydrogen chemisorption on the basal surface of VS_2_ monolayer (S2).


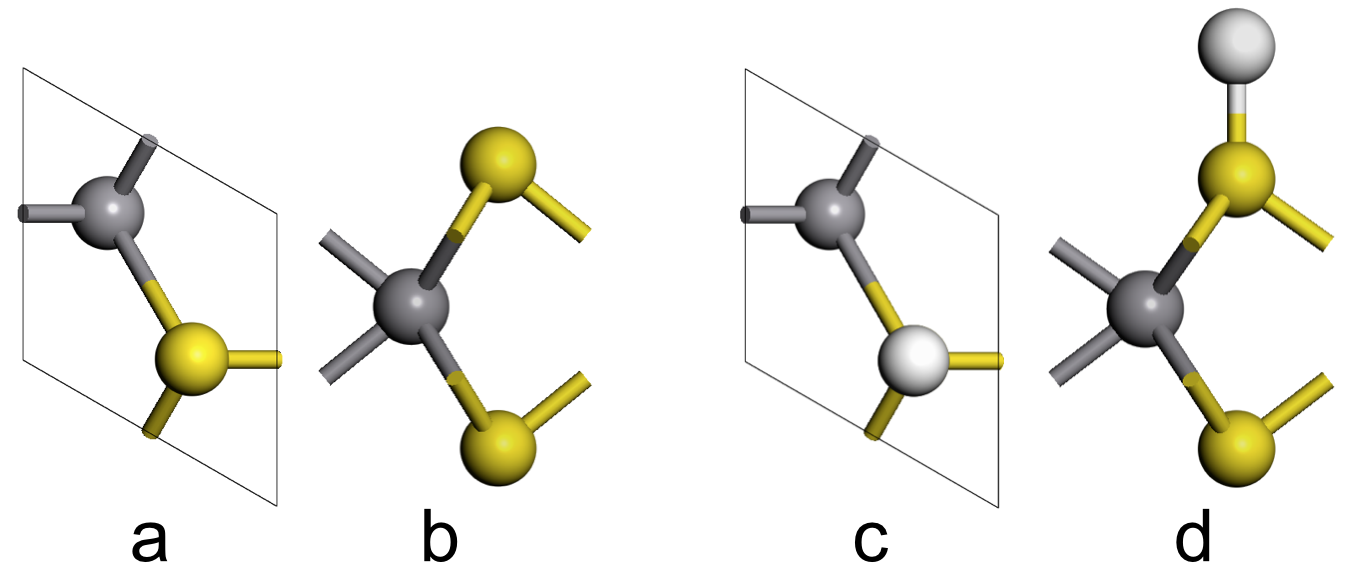


S1, **Relaxed pure VS_2_ monolayer unit cell**: (a) top view, (b) side view, relaxed VS_2_ monolayer unit cell with one side fully hydrogenated: (c) top view, (d) side view.


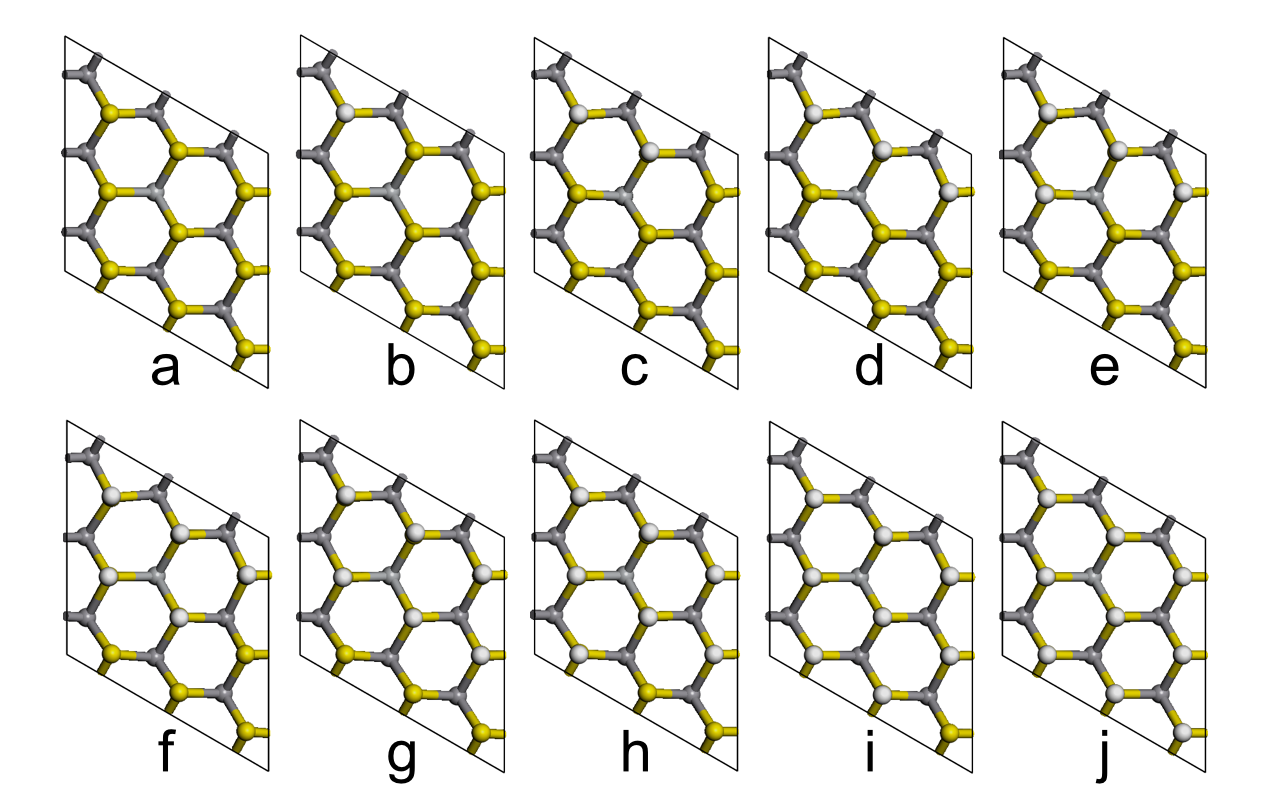


S2, **Relaxed 331 supercells of VS_2_-Ti-doped from pure to full hydrogen coverages**: (a) 0/9 or pure, (b) 1/9, (c) 2/9, (d) 3/9, (e) 4/9, (f) 5/9, (g) 6/9, (h) 7/9, (i) 8/9, (j) 9/9 or full hydrogen coverage.





S3, **Calculated overpotentials as a function of H-coverage of VS_2_ with 3 dopant (Ti, W, Ni) atoms in 331 supercells.**


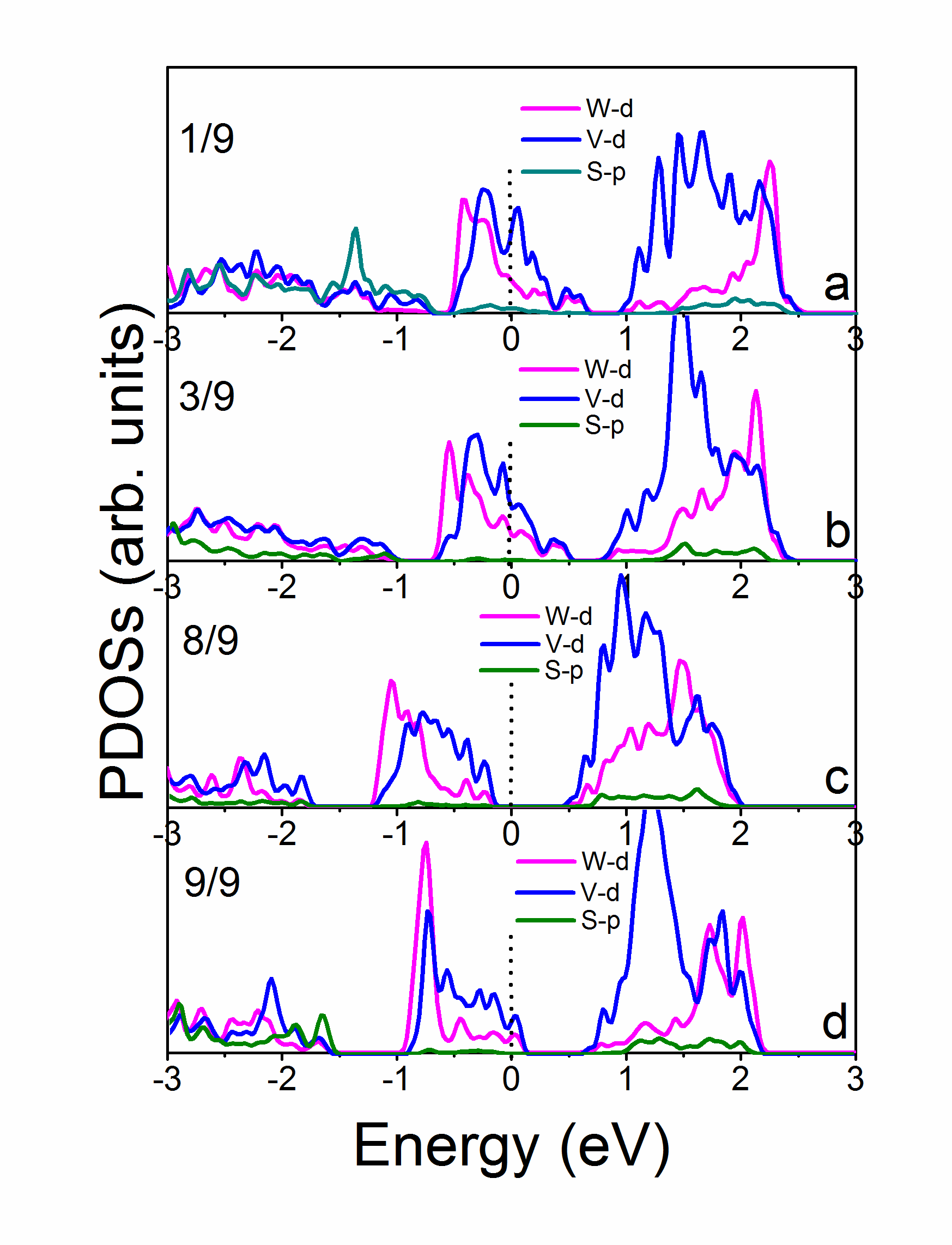


S4, **Calculated partial density of states of various H-covered VS_2_-W monolayer in 331 supercell with a hydrogen coverage** at: (a) 1/9, (b) 3/9, (c) 8/9, and (d) 9/9.


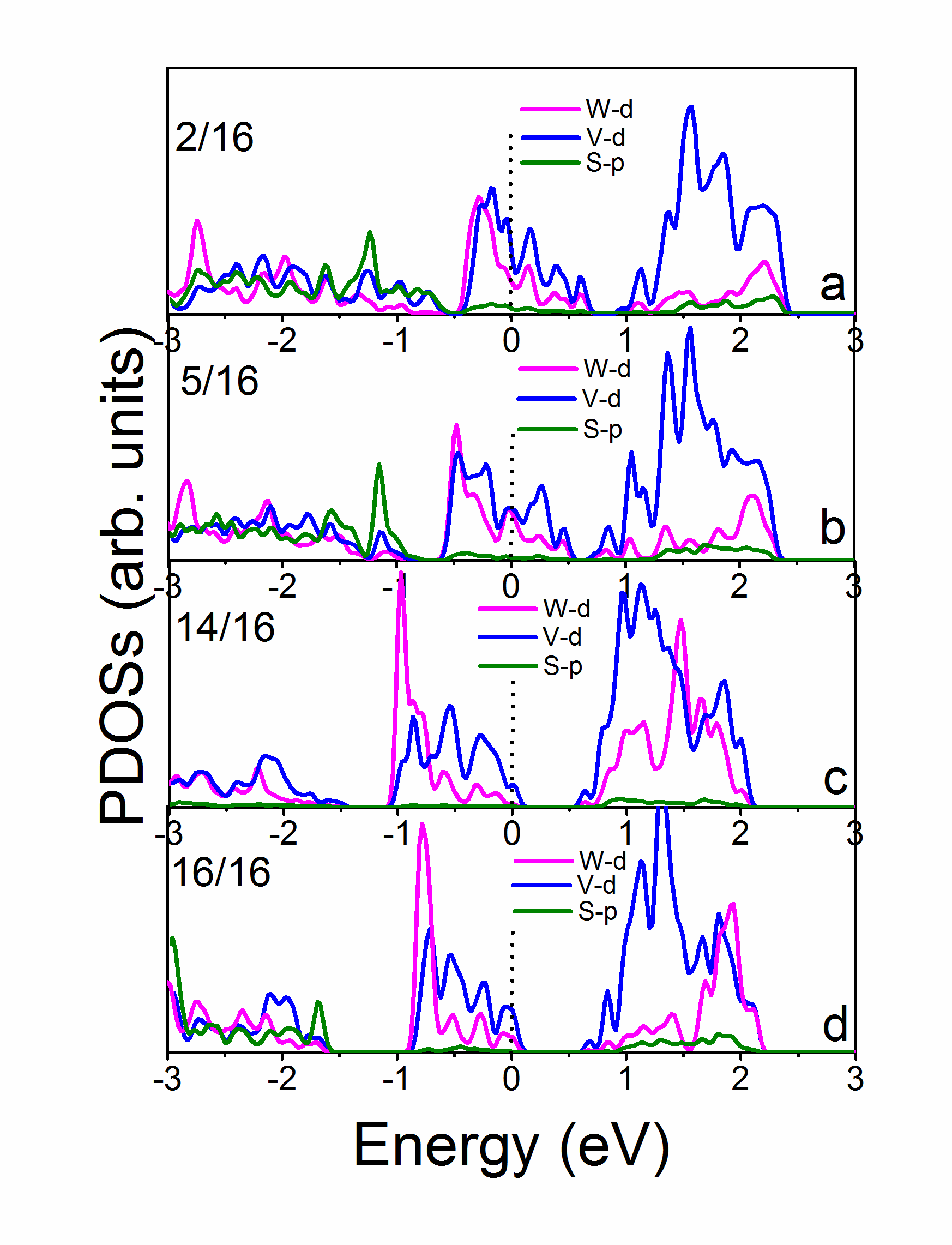


S5, **Calculated partial density of states of various H-covered VS_2_-W monolayer in 441 supercell with a hydrogen coverage** at: (a) 2/16, (b) 5/16, (c) 14/16, and (d) 16/16.


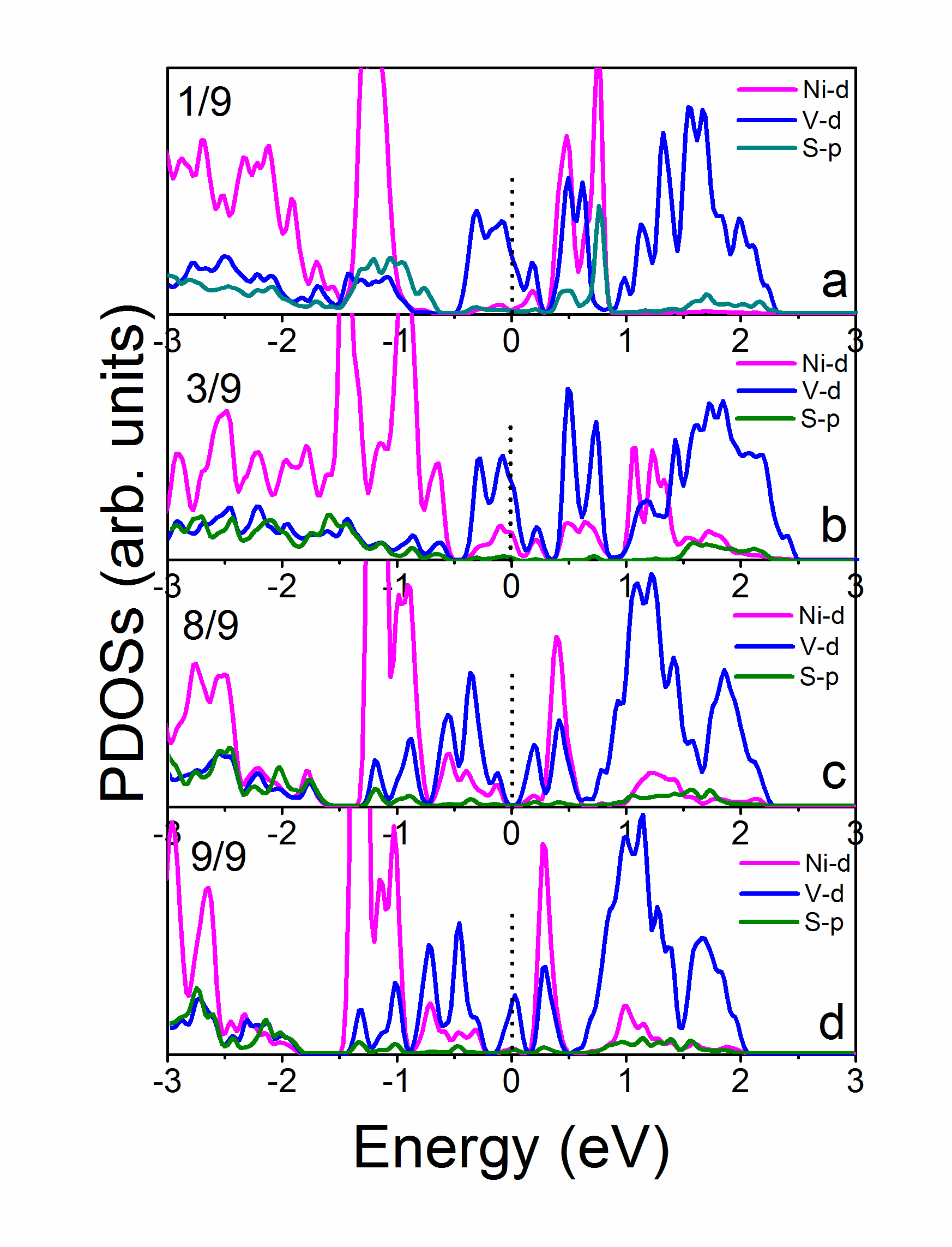


S6, **Calculated partial density of states of various H-covered VS_2_-Ni monolayer in 331 supercell with a hydrogen coverage** at: (a) 1/9, (b) 3/9, (c) 8/9, and (d) 9/9.


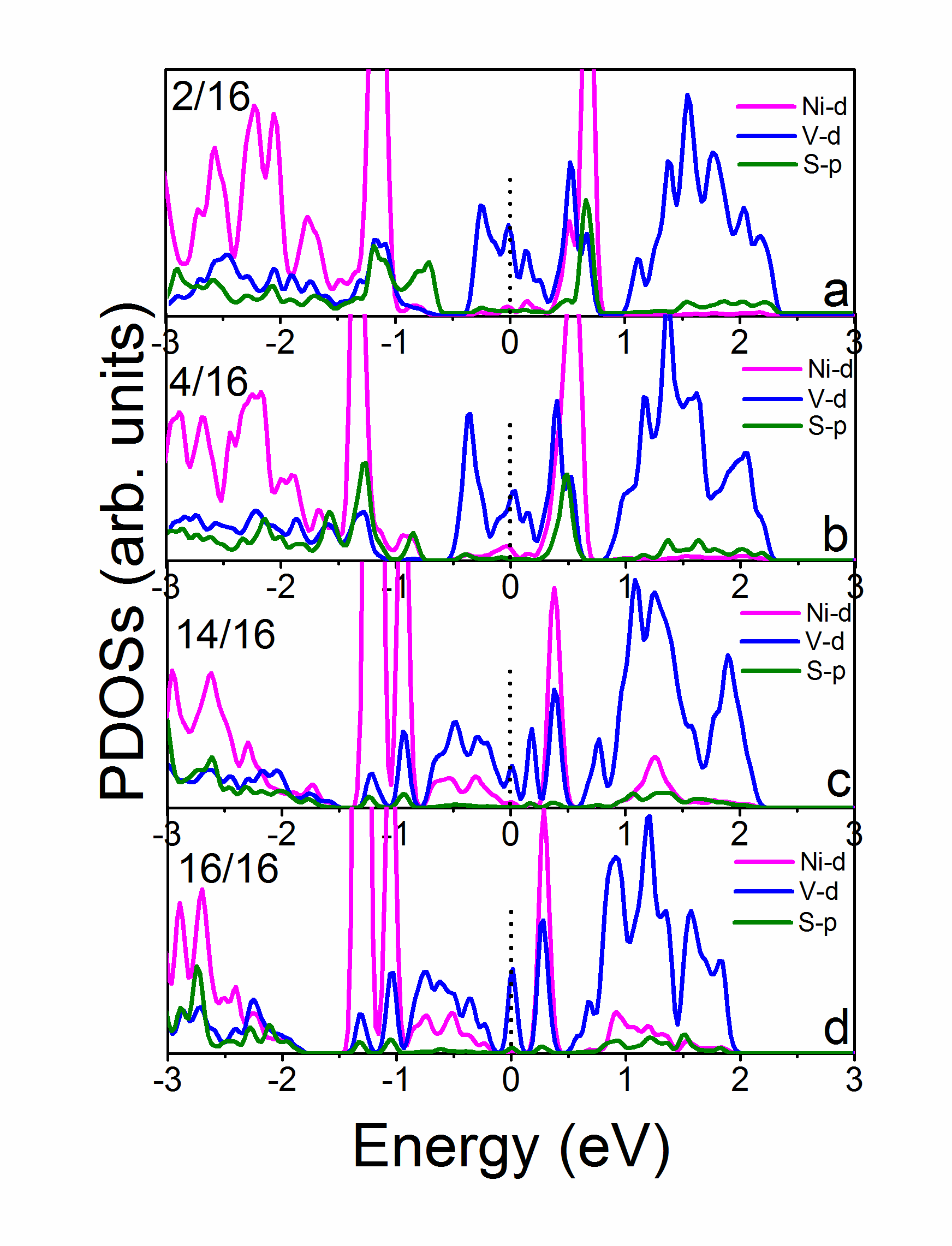


S7, **Calculated partial density of states of various H-covered VS_2_-Ni monolayer in 441 supercell with a hydrogen coverage** at: (a) 2/16, (b) 5/16, (c) 14/16, and (d) 16/16.
